# Effects of Top Management Team Characteristics on Patent Strategic Change and Firm Performance

**DOI:** 10.3389/fpsyg.2021.762499

**Published:** 2022-01-11

**Authors:** Yongtao Zhou, Yi Zhou, Li Zhang, Xu Zhao, Weijing Chen

**Affiliations:** ^1^Business School, Hubei University, Wuhan, China; ^2^Economics and Management School, Wuhan University, Wuhan, China; ^3^School of Economics and Management, Xi'an University of Technology, Xi'an, China

**Keywords:** enterprise patent strategic change, top management team characteristics, top management team emotional intelligence, top management team safety climate, firm performance

## Abstract

Patent strategy is increasingly recognized as a vital contributor in promoting core competitiveness of an enterprise. A top management team (TMT) has been indicated as one of the key factors driving changes in patent strategy. Based on upper echelons theory, this study examines how TMT characteristics, including, team diversity, emotional intelligence, and safety climate, influence enterprise patent strategic change and, hence, the business outcome. The data from 930 top managers in 228 enterprises showed that the changes in patent strategies are significantly influenced by the characteristics of top managers. These aforementioned internal TMT factors have diverse effects on the speed and scope of the enterprise patent strategic change, which in turn affects firm performance in a positive and negative way, respectively.

## Introduction

A vital factor for the economic growth and the wealth of nations, innovation-driven development in science and technology is viewed as a key national strategy in the global economic competition. As world economies are increasingly committed to becoming innovation-oriented societies, which demands enormous attention to important strategic assets, intellectual property rights, many organizations are characterized by innovation-focused development strategies and restless pursuit of appropriate patent strategies, to ensure core competitiveness and sustainable development (Zhou et al., 2009a). However, the amount and significance of patents do not guarantee success in the competition. Due to changes in both internal and external environments, enterprises are always confronted with huge challenges such as iteration of the patent technology, patent life cycle, and “winner-takes-all” patent competition (Parnell, 1998; Yang and Rui, 2010). As a former top leader in the mobile phone business, Nokia used to own much more competitive patents than other companies, whereas Huwei, the leader of the communications industry, has succeeded in maintaining its leading place in the world. Why enterprises with cutting-edge technologies and equally huge amount of patents end up so differently? From a strategic management perspective, the success is attributed to changes in the strategy in response to circumstantial variations when facing dramatic challenges.

As decision-makers and implementers, the top management team (TMT) exerts a significant influence on the structure and enforcement of strategic change (Zhou et al., 2009a). Thus, the relationship between TMT and strategic change has attracted a lot of research interest. Draw on upper echelons theory, researchers have examined how the TMT impacts company strategic change and performance, with their personal traits (Hambrick, 2007), self-portrait (Camelo Ordaz et al., 2005), team diversity (Harmancioglu et al., 2010), and safety climate perception (Cannella et al., 2008). However, it is rather questionable whether these findings also apply to a patent strategic change (PSC), a particular type of enterprise strategic change. A cross-study concept concerning both strategic change and patent strategy, PSC is originally proposed and developed by Zhou et al. (2009a). In the conceptual model demonstrating PSC formation, the effect of organizational cognition on PSC and, hence, firm performance, is mediated by the control mode, stimulated by internal and external environmental factors (Zhou et al., 2009b). Lacking empirical validation, the PSC framework underlines organizational cognition as a key influencing factor, which, however, connotes knowledge structure, core belief, and cause and effect scheme (Walsh, 1995). In fact, by contributing PSC to organization knowledge and operating inertia, the impact of TMT characteristics is overlooked. In this way, it is hard to explain why many well-developed enterprises have been suffered from wrong strategies by their top executives, who were once the founders of the enterprises. Therefore, understanding TMT characteristics may enable enterprises to better determine the changes in patent strategy and better analyze them from the perspective of an organizational structure, thus achieving a clearer and more specific understanding of the impact that organization transformation has on PSC (Wang, 2016).

The aim of the current study was to analyze the relationship among TMT, PSC, and firm performance. By empirically validating the influencing mechanism of certain organizational factors on PSC, including TMT diversity, TMT emotional intelligence, and TMT safety climate, this study offers theoretical and managerial contributions. First, it extends knowledge on PSC formation and the consequence with empirical evidence. Second, this study adds to the upper echelon literature by focusing on the patent technology-based strategic change. Third, it provides new insight into organizational cognition from the TMT perspective. Fourth, this study offers guidance for strategists on building TMTs at different developmental stages, reducing the negative impact that an individual executive personality has on implementing PSC and constructing more objective changes to better cope with fierce environmental changes and promote enterprise competitiveness.

## Literature Review and Hypothesis Development

### Patent Strategic Change of an Enterprise

The patent strategic change of an enterprise is a cross-study concept relating both enterprise strategic change and patent strategy research. Being defined as “the strategic management activity that an enterprise conducts to be constantly adapted to the organization, content, state, and portfolio of its patent strategy,” it leads the enterprise to “continuously relocate its relative resources, for the sake of gaining competitive edge and meeting changes in the dynamic business environment” (Zhou et al., 2009a, p. 21). The implementation and updating of patent strategy do not only present the innovative advances of the enterprise but also its holistic view of its patent strategy (Carpenter et al., 2004), as it suggests the systematic arrangements of different contributing parts in the whole patent strategic decision-making process (Lester, 2018).

The speed and scope of PSC of an enterprise are considered to be two dimensions of this concept (Wiersema and Bantel, 1992; Zhou et al., 2009a). PSC speed reflects how fast patent strategy advances from the original state to a new state, that is, how much time the enterprise needs to carry out the change. The major factor deciding the speed is the formulation and implementation rate of the strategy. The length of the time spent from the scanning of the patent technology to the enforcement of the new patent strategy determines the outcome of its PSC (Díaz-Fernández et al., 2019). Other factors may also influence the speed, such as the external environment, enterprise life cycle, patent life cycle, and decision-making process of top executives (Keck, 1997; Díaz-Fernández et al., 2019).

Another dimension of PSC of an enterprise is its scope, which describes the depth, breadth, and intensity of the change (Wu et al., 2018), reflecting the corporate intellectual property culture, the thoughts underpinning patent strategy, and resources committed to the strategy. The scope of the change is seen as an important enterprise response to the dynamic external environment. Some scholars maintain that the changes associated with top executives may incur intense organizational changes, whereas a relatively stable external environment may help the enterprise to achieve a greater PSC scope (Cheng, 2008; Yang and Wang, 2014).

### Characteristics of the Top Management Team and Patent Strategic Change

The existing literature is largely based on the theory of upper echelons suggested by Hambrick and Mason (1984), examining the individuals responsible for the organization and advocating positive relationships between a variety of TMT demographic indicators and firm outcomes. The theory suggests that observable characteristics of the TMT, such as age, education, or experience, are good surrogates for psychological and cognitive traits, and that they influence firm results (Camelo Ordaz et al., 2005; Harmancioglu et al., 2010).

Extant research has successfully indicated various internal and external factors affecting changes in the patent strategy, among which TMT is viewed as one of the key elements (Wang, 2016; Zhao et al., 2020; She et al., 2021). Including CEO, board of directors, independent directors, and top managers, TMT members are faced with both opportunities and challenges due to rapid changes in the industrial and economic context of enterprises. Due to inability to handle problems relying on individual strengths, a TMT composed of members with diverse professional backgrounds, skill sets, experience, and networks has become a critical approach for firms to boost businesses, develop strategies, and make progresses (Sambharya, 1996). In the process of formulating and implementing patent strategies, top executives take different roles to initiate, execute, organize, and supervise, with different responsibilities calling for a vast spectrum of knowledge, experience, skills, perspectives, and styles. These inherent individual qualities, as well as the heterogeneous team qualities, distill into the changing process of patent strategy imperceptibly (Carpenter et al., 2004; Wang, 2016). Based on a holistic perspective, personal characteristics of top executives, including age, values, educational background, working experience, emotional intelligence, and psychological safety climate, may all impact the changes in corporate strategy. Besides, other factors, such as TMT group process, TMT composition, and TMT operating process, also influence the changing course (Wang, 2016; Lester, 2018).

Diversified individual characteristics of TMT members contribute to PSCs in different ways, among which age is a significant factor (Rong and Wang, 2021). A variance in age usually means diversity in personal development, career stages, and, hence, difference in values and choices. Younger teams have more aspirations to implement strategic changes (Wiersema and Bantel, 1992). Moreover, a larger variance in age is positively associated with a higher intention to leave, since members of similar age are likely to share values and perspectives. Larger age gaps may be associated with higher levels of arguments and lower levels of trust, resulting in higher turnover rates in TMT, more hindrance to the formulation and implementation of corporate strategies, and smaller strategic changes.

Apart from age, individual educational background also plays a critical role in affecting PSC. It was found that the higher the average educational level of the team is, the more willing the members are to enforce changes in the patent strategy (Wiersema and Bantel, 1992). Another factor intrinsic to top executives, the tenure of office, is found relevant to corporate strategic changes. Longer tenures bring more opportunities to communicate and coordinate, leading to group consensus about corporate strategies. A longer tenure team is more inclined to share common ideas and perceptions, maintaining a more cohesive and stable team (Adizes, 1979). However, it may also result in conservative executives and stagnant strategies, posing problems for achieving goals of the enterprise, i.e., keeping an edge in the patent strategy. Conversely, shorter tenures mean different viewpoints and greater heterogeneity among the members, leading to better adaptation of the dynamics, more innovative strategies, new products and patents, and bigger business numbers (Keck, 1997; Wu et al., 2018).

Professional experience of individual top executives exerts influence in implementing the corporate strategy. Drawing on upper echelon theory, previous research has proved that, more exposure to international experience among team members leads to more internationally focused enterprise strategies (Sambharya, 1996). The composition of TMT, that is, the degree of homogeneity and heterogeneity in terms of demographics, cognitive traits, values, and experience, also impacts on PSC. Although controversial consequences are observed about the effects of team homogeneity and heterogeneity on decision-making and firm performance (Hurt, 2015; Zhong et al., 2019), heterogeneity is preferred as far as PSC is concerned, for the sake of product innovation and patent acquisition. As the span of specialty and various perspectives are preserved and the knowledge base of TMT is enhanced, the strategy pursued by the team is naturally expected to be even more diversified and enriched. Finally, more diversity in strategic measures and perspectives also help the enterprise stay agile and competitive in the fast changing environment (Bjornali et al., 2016).

The size of the TMT has always been an interesting research topic. Some scholars believe that expanding the team is an important means of achieving successful return on innovative investment, as the capability and resources increase when the team becomes bigger. A wider range of new perspectives and expertise, with the potential of drastically reducing the risk of hasty decision-making, is particularly critical when the enterprise is planning on a strategic transition or tapping into an unexplored territory (Yang and Wang, 2014). Controversially, it is found that a smaller TMT is more efficient in making timely decisions and overseeing the execution process. Smaller teams mean more specific responsibilities for each member and less friction inside the team, leading to a higher quality patent strategy or faster implementation of changes in the patent strategy. However, the TMT size has also been proven irrelevant to team decision power, corporate strategy, or firm performance. Enterprises with the same TMT size exhibit a completely different behavior, business performance, and innovation output (Cheng, 2008). Interestingly, however, no matter the team is expanding or downsizing, the effect on firm performance is always negative, suggesting no connection between TMT size and corporate output (Bjornali et al., 2016).

More recently, the research community has taken on interest in investigating the emotional intelligence of TMT. Emotional quotient, or EQ, indicator of emotional intelligence, refers to the ability of individuals to monitor and manage their own emotions or emotions of others, to discriminate among them, and to use this information to guide their thinking and action (Carpenter et al., 2004; Bjornali et al., 2016). The emotional intelligence of a team refers to the capability of the team in managing emotional evolution by creating shared norms, building trust, developing team identity, and boosting teamwork effectiveness. It is how the team manages emotional dynamics during teamwork (Knockaert et al., 2014). When making strategic decisions, higher TMT emotional intelligence enables the members to work in a more coherent and consistent manner, cooperate better, and communicate with more empathy, hence, producing business decisions and an intellectual output with better quality and higher efficiency. In the meantime, it is also noteworthy that emotional intelligence of the team may suppress PSC scope, as it helps unify the views of team members on the direction of corporate innovation and development, making them more inclined to promote existing superior patents to gain the competitive edge.

The subject of perception of safety climate by TMT has also been studied recently. Communicated by safety-related behavior of CEO and his/her emphasis on safety when interacting with members (Lee et al., 2017), TMT safety climate refers to the organizational environment that top managers perceive. Furthermore, the concept highlights the safety-related duty of TMT for work and responsibility. The perception of safety climate helps top executives focus on the safe working environment, which brings about higher productivity and faster changes in the patent strategy, with more technical and human resources support. However, it also means that top management would opt for more predicable changes in the patent strategy in the hope of a secure business environment, which would in turn suppress the speed or scope of changes in patent strategy. These measures make it less likely that the enterprise will increase the development of other patents, affecting PSC negatively (Daellenbach et al., 2002).

### Top Management Team Diversity, PSC Speed, and PSC Scope

Although influenced by both internal and external environments, the change of enterprise patent strategy in the current study is investigated from an internal perspective, as we adopt a TMT point of view.

The composition of TMT has received a great attention, as the team runs the company and works as the driving force of its development. Actually, TMT specialty means it composes members of different gender, age, and occupational background, for maintaining its diversity. In this sense, it provides heterogeneous knowledge for corporate developmental strategy, preventing the enterprise from making wrong strategic decisions on the basis of narrowly focused information and knowledge (Hsu and Huang, 2011; Hurt, 2015). TMT diversity has been measured mainly by the gender and age of team members, reflecting the impact of internal social classification on PSC of an enterprise. This is mainly because senior managers usually have richer working experience and heterogeneous management experience or skills. In contrast, a certain proportion of female managers often brings more vitality to the TMT. Further, however, the influencing mechanisms of TMT diversity on the speed and scope of PSC of an enterprise are different (Chuang et al., 2007). Empirical studies have shown that the diversity in gender and age of the members will lead to disagreement within the team. High TMT diversity leads to highly diversified opinions inside the team and hence impedes the decision-making progress of organizational strategies and slows down PSC speed. Disagreement among team members also leads to longer discussion time and higher discussion cost, thus decreasing the speed of PSC. Moreover, more TMT internal diversity of TMT means more conservative strategic decisions and more cautious changes in the scope and direction of patent strategy, which eventually limits the PSC scope of the enterprise (Buyl et al., 2010; Wang, 2016). Thus, we hypothesized the following:

H1a: TMT diversity negatively affects the speed of enterprise patent strategic change.
H1b: TMT diversity negatively affects the scope of enterprise patent strategic change.

### Top Management Team Emotional Intelligence, PSC Speed, and PSC Scope

The emotional intelligence of TMT is an important factor affecting the capability of the team (Zhou et al., 2020). Team members with high emotional intelligence lead to harmonious group atmosphere, powerful leadership, flexible management, and other positive influence on the corporate development (Su et al., 2021). High EQ members are more likely to reach a consensus when deciding on the changes of patent strategy. Furthermore, in the decision-making process, they tend to ponder more over the development of the enterprise, contributing new ideas and knowledge, proposing effective measures to the change of enterprise patent strategy. In the meantime, the speed of PSC is increased, and competitive edge is gained since the enterprise utilizes its own advantages more flexibly to carry out and enhance PSC (Wiersema and Bantel, 1992; Auh and Menguc, 2005; Gauthier et al., 2019). As to the scope of the change of the patent strategy, top managers with high EQ are prone to more unified and similar opinions. Naturally, not being divided by different orders from management, the changes in patent strategies become more consistent. As the PSC scope decreases, the competitive edge is attained with the enterprise developing its own superior patents. Thus, we hypothesized the following:

H2a: TMT emotional intelligence positively affects the speed of enterprise patent strategic change.
H2b: TMT emotional intelligence negatively affects the scope of enterprise patent strategic change.

### Top Management Team Safety Climate, PSC Speed, and PSC Scope

The safety climate of TMT refers to organizational environment influenced by two factors, namely, safety-related behaviors of the CEO and emphasis of the CEO on safety when interacting with TMT (Lee et al., 2017; Richard et al., 2019). It highlights the duties of TMT to ensure safety in work and responsibilities within the enterprise. A highly safe climate perceived by top executives helps improve the professional self-identity of employees and their recognition of care and support from the organization. As a result, the enterprise performance and output are increased (Zhang and Zhu, 2021). In a highly safe climate, top managers are more likely to focus on internal safety environment and provide support to improve working environment, resulting in better working conditions and higher working efficiency. Besides, the speed of PSC of an enterprise is accelerated with more technological support to the patent strategy (Gauthier et al., 2019). Meanwhile, however, high demands on safety climate make the team more conservative in exploring new businesses, which decreases the scope of the change in enterprise patent strategy and reduces the likelihood of promoting other patent research (Wiersema and Bantel, 1992). Thus, we hypothesized the following:

H3a: TMT safety climate positively affects the speed of enterprise patent strategic change.
H3b: TMT safety climate negatively affects the scope of enterprise patent strategic change.

### Patent Strategic Change Speed, PSC Scope, and Firm Performance

Improving performance is believed to be at the core of the corporate development. When competing in the patent market, enterprises may adjust their patent strategy to gain a bigger market share, foster product innovation, and hence, achieve their performance objectives (Lampaki and Papadakis, 2018; Medina et al., 2018). However, PSC speed and PSC scope may influence the performance along separate paths and from different directions.

As one of the most concerned indicators in the evaluation of an enterprise, performance is affected by the speed of PSC of an enterprise. Faster changes in patent strategy, aiming to provide the firm with more rapid and effective patent strategic response in the process of innovation and development, enable the enterprise to be more advantageous in the product innovation market and more competitive in the new product market, resulting in better performance (Yokota and Mitsuhashi, 2008). Moreover, acceleration of PSC helps to improve the strategic reaction of an enterprise effectively against the market change, developing its patent strategy in a market-oriented manner. Rapidly changing patent strategy provides the firm with more powerful patent competitiveness, promotes its core business, and improves its performance (Wangrow et al., 2014).

However, enlarging the scope of PSC of an enterprise means that the enterprise develops in different innovative directions. Excessive changes in patent strategy may lead the enterprise into more product markets, with various patent strategy demands. Since the resources supporting patents are fixed and limited within an enterprise, scattering them over an increased scope of patent changes may lead to failure in keeping a relatively competitive position in the product market (Sambharya, 1996; Awino and Francis, 2018). As a result, market share is not likely to increase and firm performance is negatively affected. Moreover, broadening the scope of PSC means more market expansion cost and more resources to invest for maintaining competitiveness (Medina et al., 2018). In fact, firm performance is often negatively affected by the enlarged scope of patent strategy change. Thus, we proposed the following hypotheses:

H4a: The speed of enterprise patent strategic change positively affects enterprise performance.
H4b: The scope of enterprise patent strategic change negatively affects enterprise performance.

### Conceptual Model

**Figure 1:**
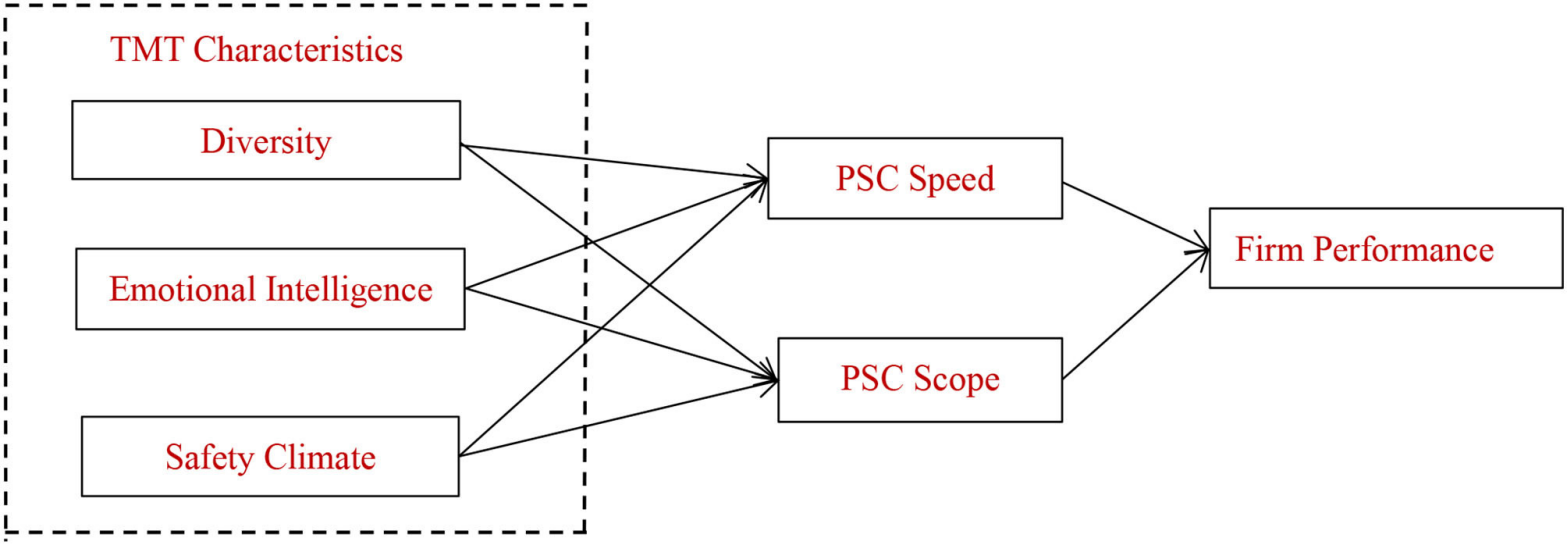
Conceptual model. TMT, top management team; PSC, patent strategic change.

To understand the formation of PSC of an enterprise and its role in the business performance, we adopted an upper echelon theory perspective (Hambrick, 2007). Based on the idea that top executives view their situations through their own highly personalized lenses, researchers have attributed variations in strategic viewpoints to the differences among executives in their experiences, values, personalities, and other human factors. The degrees of homogeneity and heterogeneity in terms of demographics, cognitive traits, values, and experience impact the strategic change. Accordingly, different compositions of TMT play a role in the formation and implementation of the patent strategy (Chen et al., 2010; Wu et al., 2018). We anticipated that TMT diversity, TMT emotional intelligence, and TMT perception of safety climate serve as relevant antecedents of PSC, which in turn is a significant driver of firm performance (Figure 1).

## Methods and Results

### Data Collection

The data collection and analysis were carried out to verify the influencing factors of PSC of an enterprise. We used team diversity, TMT emotional intelligence, and TMT safety climate to capture the impact of TMT characteristics on PSC, and further, to test the relationship between the speed and scope of the change and firm performance, we conducted surveys in MBA and EMBA classes and numerous enterprises, where managers were required to complete the questions in the classroom or in their workplaces. We based our questionnaire on mature scales from domestic and foreign researches, with some necessary modifications on certain items according to the actual situation. All scales were translated and back-translated between Chinese and English by bilingual translators to ensure accuracy. The survey process lasted 10 months from March to November 2017, and the target audience consisted top executives in the high-tech innovation industry from Hubei, Shanxi, Henan, and Sichuan provinces in China. Respondents completed questions about PSC, TMT, and firm performance, reflecting actual situations within the firm. The data were collected from 10 industries, including electronics manufacturing, aerospace power, communications engineering, and mechanical design. A total of 1,292 questionnaires were delivered in 303 enterprises, among which 930 usable responses from 228 enterprises were obtained, resulting in an effective response rate of 71.98%. The respondent profile is shown in Table 1.

**Table 1:** Descriptive statistics.

**Types**	**Samples**	**Ratios**
**Enterprise types (*****N*** **=** **76)**		
State-owned enterprise	105	45.05%
Private enterprise	45	19.74%
Foreign enterprise	51	22.37%
Others	27	11.84%
**Enterprise history**		
**Years**		
0-5	41	17.90%
6-10	54	23.68%
11-15	35	15.35%
>15	98	42.98%
**Enterprise development stage**		
**Stages**		
Initial stage	27	11.84%
Developing stage	74	32.46%
Expanding stage	58	25.44%
Mature stage	60	26.32%
Recessing stage	9	3.95%
**Enterprise size**		
**Number of people**		
<50	27	11.84%
50-100	46	20.18%
101-200	110	48.25%
201-500	29	12.72%
>500	16	7.02%

### Measurements and Results

Constructs in the present study include TMT diversity, TMT emotional intelligence, TMT safety climate, PSC speed, PSC scope, and firm performance. Measurements were completed using a 7-point Likert scale anchored by 1 = strongly agree and 7 = strongly disagree.

(1) PSC of an enterprise is measured along two dimensions, namely, the speed and the scope of PSC. Six items were employed, including “The speed of enterprise adjusting patent strategy, to tackle its competitors' marketing movements” (measuring the speed) and “The range of newly patented technological products is continuously expanding” (measuring the scope), as derived from the scale developed by Gu (2008) and Zhou et al. (2009a) (Cronbach's alpha = 0.79).

(2) TMT diversity is assessed along the dimensions of age and gender of the team members (Liang et al., 2007), since the increase of age means richer and more heterogeneous management experience, and the difference in gender often leads to more vitality among the team.

(3) TMT emotional intelligence assesses the capability of the managers to capture and control emotions and emotion-related psychology and behavior in work and communication. Sixteen items, such as “creating opportunities actively” and “not easily aroused to anger,” were included based on the scale developed by Zhang and Ling (2008), with internal consistency coefficient assessed reliability of score within group (RWG) = 0.82, intraclass correlation coefficient (ICC) (1) = 0.13, ICC (2) = 0.42 (Cronbach's alpha = 0.93).

(4) TMT safety climate taps into the consciousness of safety of the top managers in organizational behaviors. It was measured based on the scale developed by Tucker et al. (2016), including ten items like “Listen carefully to employees' opinions on safety,” with the internal consistency coefficient assessed RWG = 0.93, ICC (1) = 0.13, ICC (2) = 0.44 (Cronbach's alpha = 0.86).

(5) Firm performance was typically measured by five items including “compared with major competitors, the profit of the company is very high,” derived from the scale developed by Jia et al. (2013) (Cronbach's alpha = 0.79).

These scales show strong reliability as indicated by Cronbach's alpha. The value for each scale was above 0.7, proving reliability for each variable. We presented the descriptive statistics and correlation matrix in Table 2. The correlation coefficients between the variables were either low or medium, excluding multicollinearity issues.

**Table 2:** Construct correlations.

**Variables**	**Mean**	**SD**	**1**	**2**	**3**	**4**	**5**	**6**	**7**	**8**	**9**
Enterprise size	6.41	1.51									
Enterprise history	24.43	23.48	0.16								
Enterprise development stage	3.19	0.45	0.26^*^	0.02							
PSC speed	5.15	0.67	−0.32^**^	0.13^*^	0.29^*^	0.65					
PSC scope	5.31	0.42	−0.04	−0.22^*^	−0.07	0.12	0.70				
TMT emotional intelligence	5.52	0.72	0.04	0.19^*^	0.17^*^	0.26^*^	−0.28^*^	0.79			
TMT safety climate	5.84	0.68	0.23	0.14	0.11	0.15^*^	−0.20^*^	0.33	0.81		
TMT diversity	4.33	0.68	0.10	−0.15	−0.33	−0.21	0.09	−0.13	−0.20	0.66	
Firm performance	5.93	0.98	−0.10	0.08	0.21	0.12^*^	−0.15^*^	0.09	0.35	−0.21	0.85

* *p < 0.05 and*

** *p < 0.001. TMT, top management team; PSC, patent strategic change*.

This study mainly discusses the impact of TMT characteristics on firm performance through the intermediary path of the patent strategy change, thus not directly testing the impact of TMT characteristics on firm performance. An SPSS regression was performed, and the results are shown in Table 3. In Model 1, only control variables were added to verify their influence on the speed of the PSC of an enterprise. Furthermore, TMT diversity was added in Model 2 to prove its relationship with the speed of the PSC of an enterprise. The results show a significant negative correlation between them (β = 0.30, *p* < 0.05), supporting H1a. In Model 3, TMT emotional intelligence was added, and it was found to be significantly positively correlated with the speed of the PSC of an enterprise (β = 0.31, *p* < 0.05), supporting H2a. Model 4 validates the relationship between the TMT safety climate and the speed of the PSC of an enterprise. The regression analysis shows a significant positive correlation between them (β = 0.15, *p* < 0.01), thus H3a is supported.

**Table 3:** Regression analysis: Effects on PSC speed.

**PSC speed**				
**Variable**	**Model 1**	**Model 2**	**Model 3**	**Model 4**
Intercept	5.52	5.35	6.42	2.22
Enterprise size	−0.31^*^	−0.32	−0.29	−0.32
Enterprise development stage	0.14^*^	0.31^*^	0.31^*^	0.29^*^
TMT diversity		−0.30^*^		
TMT emotional intelligence			0.31^*^	
TMT safety climate				0.15^**^

* *p < 0.05 and*

** *p < 0.001*.

In Table 4, Model 6 tested the relationship between the TMT diversity and the scope of the PSC of an enterprise. Results indicate no correlation (β = 0.1, *p* > 0.1), not supporting H1b. In Model 7, TMT emotional intelligence was added, and it was found to have no significant correlation with the scope of the PSC of an enterprise (β = −0.33, *p* > 0.1). Thus, H2b is not supported. Model 8 validated the relationship between the TMT safety climate and the scope of the PSC of an enterprise. The regression analysis shows a significant negative correlation between them (β = −0.22, *p* < 0.01), thus supporting H3b.

**Table 4:** Regression analysis: Effects on PSC scope.

**PSC scope**				
**Variable**	**Model 5**	**Model 6**	**Model 7**	**Model 8**
Intercept	5.21	5.03	5.95	3.35
Enterprise size	−0.06	−0.07	−0.03	−0.02
Enterprise development stage	−0.08^*^	−0.07^*^	−0.08^*^	−0.07^*^
TMT diversity		−0.11		
TMT emotional intelligence			−0.33	
TMT safety climate				−0.22^**^

* *p < 0.05 and*

** *p < 0.001*.

Table 5 shows that the speed of the PSC of an enterprise is significantly positively correlated with firm performance (β = 0.14, *p* < 0.05), and the scope of the PSC of an enterprise is significantly negatively correlated with performance (β = −0.18, *p* < 0.01). Thus, H4a and H4b are supported.

**Table 5:** Regression analysis: Effects on firm performance.

**Firm performance**		
**Variable**	**Model 9**	**Model 10**
Intercept	3.61	2.79
Enterprise size	−0.11	−0.09
Enterprise development stage	0.09^*^	0.08^*^
PSC speed	0.14^*^	
PSC scope		−0.18^**^

* *p < 0.05 and*

** *p < 0.001*.

## Discussion

### Theoretical and Practical Implications

The purpose of this study was to analyze the effects of characteristics of the TMT on the PSC of an enterprise and firm performance. Changes in patent strategies indicate the innovative advances of an enterprise and are closely associated with its business performance. Extant literature provides little evidence of how TMT has influenced PSCs. Drawing on the upper echelon perspective, we proposed and empirically validated the diverse effects of TMT internal factors (i.e., team diversity, emotional intelligence, and safety climate) on the speed and scope of the strategic change of an enterprise. Also, this research tested the impact of the PSC on firm performance. Further, it confirmed that the speed and scope of the change influence the performance in a positive and negative way, respectively.

Our study provides both theoretical contributions and managerial insights. First, it adds to the limited literature that addresses the PSC of an enterprise, a novel cross-research area involving both strategic change and patent strategy studies. Besides establishing a conceptual framework from an internal perspective, our study is the first to empirically investigate the influencing factors and outcome of PSC. Also, our results demonstrate the significant driving force of TMT in causing changes in both the PSC speed and scope. Second, our study contributes to upper echelons theory by focusing on the impact of TMT on one particular aspect of strategic outcomes, patent strategies. One idea central to the theory is that “strategic decisions typically involve multiple executives” and organization outcomes are usually “the result of group decision processes” (Hambrick, 2016, p. 1). By explicitly hypothesizing and testing the relationship among TMT characteristics, PSC, and firm performance, we complemented prior upper echelons research that provides insights for strategies only in a general sense. Third, our findings affirm the contributing roles of TMT in the strategic change of an enterprise and, specifically, in the PSC (Rajagopalan and Spreitzer, 1997). Providing a theoretical foundation of predicting patent decisions, this study employs three intangible elements of TMT characteristics, namely, TMT diversity, TMT emotional intelligence, and TMT safety climate as antecedents to the speed and scope of PSC. Fourth, we explored the relationship between the scope of strategic changes and business performance. The scope of strategic changes reflects the strength of enterprise managers to adjust their strategies according to environmental changes. A wider range of strategic changes is usually accompanied by a longer period of time, bringing greater risks to enterprise managers and making it difficult to predict the firm performance after strategic changes (Haynes and Hillman, 2010). Therefore, a significant negative relationship is observed between the PSC scope and business output. Last, our findings suggest an insignificant negative association between the team diversity, emotional intelligence, and the scope of PSC, different from the traditional research conclusions. Thus, more thorough investigation into TMT as driving factors in the strategic change is necessary.

Our study also provides helpful managerial implications for enterprise patent strategists. First, faster PSCs mean more likelihood of a leading place in the patent market, thus more effectively improving the firm performance. In contrast, a broader scope of PSC means diversifying innovative resources into multiple patent markets, resulting in less competitiveness in a short period of time. Balancing the speed and scope of PSC with enterprise development is therefore of vital importance for decision-makers. Second, in the process of selecting and appointing TMT members, reducing team diversity, engaging high-emotional intelligence members, and increasing perception of safety climate offer more reliable support in decision-making and help improve interpersonal relations, resulting in more effective strategic policies. Finally, as directors of PSC, TMT members are expected to have a certain patent technological background. The scope of strategic change refers to variation in the depth, width, and size of enterprise strategic contents and difference in products, service, and target market when resources are allocated (Yi et al., 2007). As patent strategies are based on licensed technologies, featuring distinctive technological and professional expertise, PSC is therefore more definite and directive compared with strategic changes in a general sense. Hence, enterprises should take the patent technological background into consideration when building TMTs.

### Limitations and Future Research

The present study is an empirical investigation into the mechanism of TMT on the PSC of an enterprise. However, the influencing factors that are studied in this article are actually limited to TMT diversity, TMT emotional intelligence, and TMT safety climate perception. Future research could address this issue from external perspectives, such as patent environment, firm age, government financial subsidy, national policy environment, public development environment, and external competitor strategy. Research on internal factors could extend to members of the board, human capital, and social capital of the board.

There are some limitations in this study, too. As the data collected in the current study have included only limited regions of the country, the findings may not be universally applicable to all enterprises when considering patent strategies. Another limitation lies in the collection of cross-sectional data, which may result in the lack of dynamic context in the study of the scope of PSC. Future research could further explore through longitudinal data, considering that the scope of PSC is more likely to be associated with vertical research. Besides, as the sample size remains limited, future research could be conducted with a combination of first-hand and second-hand data. Finally, only internal factors have been employed, and the multiple linear regression analysis is adopted. Future research could consider incorporating certain external factors as control variables and using structural equation modeling to test the effects of TMT on PSCs.

## Data Availability Statement

The original contributions presented in the study are included in the article/supplementary materials, further inquiries can be directed to the corresponding author/s.

## Ethics Statement

The studies involving human participants were reviewed and approved by the Hubei University. The patients/participants provided their written informed consent to participate in this study.

## Author Contributions

YoZ developed the research model, analyzed the data, and co-drafted the manuscript. YiZ collected the data and co-drafted the manuscript. LZ and WC edited the manuscript. XZ analyzed the data and edited the manuscript. All authors contributed to the article and approved the submitted version.

## Funding

This work was supported by the Young Program of the State Social Science Fund: A Study of the Change of Patent Strategy in the Dynamic Environment (11CGL002); the General Program of National Natural Science Foundation of China (NSFC): The Optimization Research on the Patent Authorization in the Censor Control of time Lag (71473095). Hubei Center for Studies of Human Capital Development Strategy and Policy, and Key Research Base of Humanities and Social Science of Hubei Province.

## Conflict of Interest

The authors declare that the research was conducted in the absence of any commercial or financial relationships that could be construed as a potential conflict of interest.

## Publisher's Note

All claims expressed in this article are solely those of the authors and do not necessarily represent those of their affiliated organizations, or those of the publisher, the editors and the reviewers. Any product that may be evaluated in this article, or claim that may be made by its manufacturer, is not guaranteed or endorsed by the publisher.
